# Determinants of oral health among Iranian soldiers: a structural equation modeling study

**DOI:** 10.1186/s12903-024-05052-5

**Published:** 2024-10-25

**Authors:** Morteza Banakar, Akram Ghannadpour, Arghavan Behbahanirad, Hassan Joulaei, Kamran Bagheri Lankarani

**Affiliations:** 1 Health Policy Research Center, Institute of Health, Shiraz University of Medical Sciences, Shiraz, Iran; 2 School of Dentistry, Tehran University of Medical Sciences, Tehran, Iran; 3 Department of Dental Public Health, School of Dentistry, Shiraz University of Medical Sciences, Shiraz, Iran

**Keywords:** Oral health, Simplified oral hygiene index, Dental caries, Periodontal health, Structural equation modeling

## Abstract

**Background:**

Military personnel often face unique challenges in maintaining optimal oral health. This study investigated the oral health status, caries experience, and associated factors among a sample of Iranian soldiers, employing a structural equation modeling (SEM) approach to explore the complex interplay of socioeconomic and behavioral determinants.

**Methods:**

A cross-sectional study was conducted among 658 male soldiers aged 18–30 years from three military barracks in Fars province, Iran. Data were collected through a structured instrument and clinical oral examinations. The study employs the DMFT index, which measures caries experience based on decayed, missing, and filled teeth, along with the Simplified Oral Hygiene Index (OHI-S) to assess overall oral health status. Structural equation modeling was employed to analyze the complex relationships between socioeconomic factors, oral health behaviors, and oral health outcomes.

**Results:**

The mean DMFT score was 3.57 ± 5.91, and the mean OHIS score was 0.56 ± 1.42. SEM analysis revealed that socioeconomic status (SES) indirectly influenced DMFT and oral hygiene scores, mediated by drug use, oral hygiene practices, dietary sugar consumption, and dental visit frequency. Lower toothbrushing frequency was significantly associated with higher DMFT (Estimate = -0.064, *p* < 0.001) and OHIS scores (Estimate = -0.637, *p* < 0.001). Drug use (smoking, qalyan, alcohol) was linked to poorer oral health outcomes, while more frequent dental visits were associated with lower DMFT and OHIS scores.

**Conclusion:**

This study reveals the complex interplay between socioeconomic conditions, oral health behaviors, and oral health outcomes among Iranian soldiers. The findings highlight the need for targeted interventions to address modifiable risk factors and improve access to preventive dental care within military settings. Future longitudinal studies are warranted to elucidate further the causal pathways between these factors and oral health outcomes in military populations.

**Supplementary Information:**

The online version contains supplementary material available at 10.1186/s12903-024-05052-5.

## Background

Oral health is integral to overall well-being, reflecting physiological, social, and psychological aspects essential for quality of life [1]. Furthermore, a bidirectional relationship exists between oral and general health [2]. Despite advances in dental care and preventive measures, the global burden of oral diseases affects individuals of all ages and socioeconomic strata. According to the WHO’s 2022 Oral Health Status Report, nearly half of the global population (45% or 3.5 billion people) experienced oral diseases, with 3 out of every four affected people living in low- and middle-income countries [3]. Among these populations, military personnel represent a unique cohort with specific oral health concerns due to their distinctive lifestyle, occupational hazards, and varying access to dental care [4].

Soldiers are a high-risk group for developing oral diseases due to the demanding physical and psychological nature of military training and lifestyle. The lack of routine, challenging work environments, particularly during training, unbalanced diets, inconsistent hygiene practices, and stress further emphasize the need for adequate oral healthcare in this population [5]. Studies conducted among military personnel in various countries have consistently reported high prevalence rates of dental caries and other oral health issues [6–8].

In Iran, military service is mandatory for all healthy men for a period of 18–24 months, typically commencing at the age of 18. This mandatory service draws individuals from diverse socioeconomic backgrounds across the country. Diverse socioeconomic backgrounds make this group an interesting population for studying oral health and its determinants. However, comprehensive investigations into the oral health status, caries prevalence, and associated factors among Iranian soldiers are limited, highlighting a crucial gap in the literature [9]. The complex interplay of factors influencing oral health outcomes necessitates a multifaceted approach to analysis. Structural equation modeling (SEM) offers a sophisticated statistical technique that allows researchers to examine complex relationships between multiple variables simultaneously, providing a more holistic understanding of the factors contributing to oral health status [10]. In this study, we considered socioeconomic status (SES) as a latent variable, given its multidimensional nature and the need to account for measurement errors across various indicators.

This study uses a structural equation modeling approach to investigate oral and dental health, caries experience, and related factors in a sample of Iranian soldiers. This advanced analytical method seeks to elucidate the direct and indirect relationships between various demographic, behavioral, and environmental factors and oral health outcomes. The findings of this research will not only contribute to the existing body of knowledge on military oral health but also inform targeted interventions and policy decisions to improve the oral health status of soldiers. Moreover, this study’s results may have broader implications for understanding oral health patterns in young adult populations undergoing mandatory military service in similar contexts. By identifying key risk factors and their interrelationships, we aim to provide valuable insights that can guide the development of effective preventive strategies and oral health promotion programs within military settings and beyond.

## Methods

### Setting and study population

This cross-sectional analytical study was conducted among 658 male soldiers aged 18–30 years from three military barracks in Fars province, Iran. A multi-stage cluster sampling approach was employed. First, three military barracks in Fars province were randomly selected. Within each barracks, soldiers were then randomly sampled to participate. The recruitment took place between 2022 and 2023. We employed a systematic random sampling method to ensure a random selection of soldiers within each barracks. A list of all soldiers in each selected barracks was obtained, and every nth soldier was chosen to participate, where n was determined based on the total number of soldiers and the required sample size. This method ensured that each soldier had an equal chance of being selected, thereby minimizing selection bias.

As military service is mandatory for most young men in Iran, and duty stations are assigned randomly, the sample can be considered representative of the broader population. Ethical approval for the study was obtained from the relevant committee (IR.SUMS.DENTAL.REC.1400.066). Participation was voluntary, and all participants were informed about the study’s purpose, ensuring informed consent and confidentiality of their data. Questionnaires were assigned unique identifiers to maintain anonymity, and no personal identifying information was collected.

#### Inclusion criteria

- Male soldiers undergoing mandatory military service.
- Age 18–30 years old at the time of data collection.
- Provision of informed consent to participate in the study.

#### Exclusion criteria

- History of serious systemic diseases that could independently impact oral health, such as uncontrolled diabetes or immune deficiencies. While such conditions are rare among military conscripts, this criterion was included to ensure the validity of our clinical assessments.
- Current use of medications known to significantly affect oral health (e.g., chemotherapy, radiation therapy, long-term corticosteroid use). Although unlikely in this population, this criterion was included to account for any unforeseen cases.
- Inability to participate in the oral examination due to physical or cognitive limitations. This criterion was meant to exclude individuals whose oral health could not be accurately assessed due to physical disabilities or cognitive impairments, though such cases are rare among military personnel.

These exclusion criteria were included as a precaution to ensure that any potential confounding factors that could significantly alter oral health outcomes were accounted for. While the likelihood of such conditions in military conscripts is low, we adopted a comprehensive approach to maintain the study’s internal validity.

### Sample size calculation

The sample size was calculated using the Cochran formula [11] based on an estimated caries prevalence of 30%, a margin of error of 5%, and a confidence level of 95%. The minimum required sample size was determined to be 323. However, we opted for a larger sample to ensure sufficient statistical power for the structural equation modeling analysis, account for possible non-responses, and obtain a more representative sample. To achieve this, we applied a design effect [12] of 2, resulting in a total sample size of 646. To further strengthen the study, we recruited a total of 658 participants, all of whom participated, yielding a 100% response rate.

### Data collection

Data collection involved two components:

#### Structured instrument

Data were collected using a structured questionnaire developed based on a thorough literature review and expert consultations. A panel of experts in dental public health and community medicine ensured the content validity of the instrument (Appendix 1). A pilot study with 20 soldiers was conducted to test the clarity and relevance of the questionnaire items. The instrument was designed to collect data on.

- **Demographic characteristics**: Age, marital status, place of residence, education level, housing status, parental education levels, previous insurance coverage, employment status prior to service, birth order, and ethnicity.
- **Self-reported oral hygiene practices and Dietary habits**: Toothbrushing frequency, flossing habits, frequency of dental visits, Frequency of sugary food and drink consumption. This part incorporated validated and reliable scales and questions adapted from previously published studies on oral health behaviors and determinants [13–15].
- **Drug use**: Smoking (cigarettes), water pipe (qalyan) use, and alcohol consumption.

#### Clinical oral examinations

A trained and calibrated dentist conducted oral examinations at the military barracks using standard equipment (dental chair, headlight, mouth mirror, and probe). Intra-examiner reliability was assessed by re-examining 10% of participants, with a kappa score of 0.85 indicating good reliability. Examinations were performed according to World Health Organization (WHO) diagnostic criteria and guidelines [16]. The following indices were used.

- **Decayed**, **Missing**, **and Filled Teeth (DMFT) Index**: Assessed caries experience [16].
- **Simplified Oral Hygiene Index (OHIS)**: Evaluated oral hygiene based on the Debris Index (DI) and Calculus Index (CI) (Table 1). The OHIS assesses six surfaces on four posterior teeth (maxillary right first molar (16), maxillary left first molar (26), mandibular left first molar (36), and mandibular right first molar (46)) and two anterior teeth (maxillary right central incisor (11) and mandibular left central incisor (31)) [17].

**Table 1:** Simplified oral hygiene index (OHIS)

Scoring criteria	Calculus index	Debris index
0	No calculus present	No debris or stains are present
1	Supragingival calculus is present but covers less than 1/3 of the exposed tooth surface.	Soft debris is present but covers less than 1/3 of the tooth surface, or extrinsic stains are present without regard to the surface covered.
2	Supragingival calculus is present, covering less than 1/3 of the exposed tooth surface, but more than 1/3 of the teeth are not covered, or the presence of subgingival calculus around the neck of the tooth or both.	Soft debris covers more than 1/3 of the tooth surface but less than 2/3 of the exposed tooth surfaces.
3	Supragingival calculus is present, covering more than 2/3 of the exposed tooth surface; a heavy continuous band of subgingival calculus is around the neck of the tooth, or both.	Soft debris covers more than 2/3 of the exposed tooth surfaces.

### Statistical analysis

**Fig. 1:**
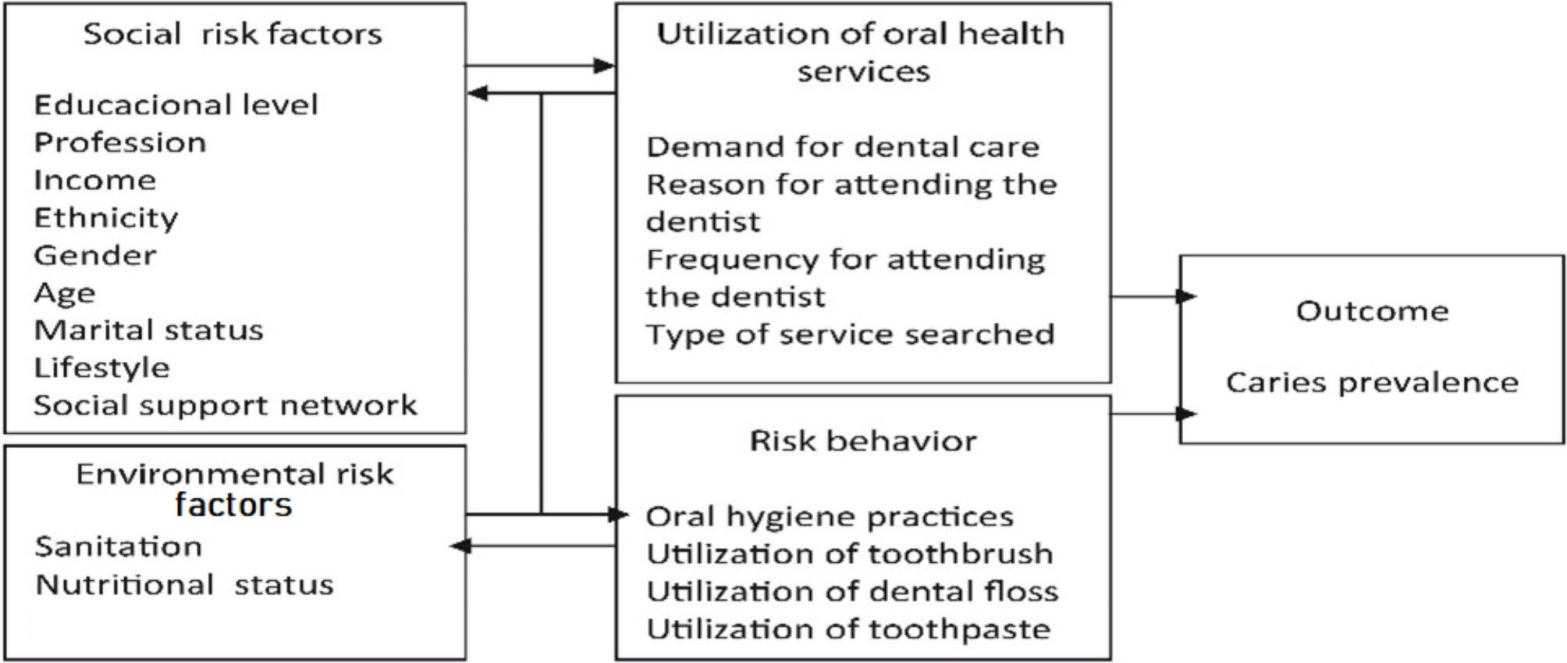
The model was adapted from Petersen’s shows Distal and proximal risk factors for dental caries analysis [18]

Descriptive statistics were performed using SPSS v20. Variables were categorized into distal and proximal factors and oral health outcomes. Structural equation modeling (SEM) was employed to analyze the complex relationships between socioeconomic factors, oral health behaviors, and oral health outcomes. In SEM, we distinguish between reflective and formative constructs. Reflective constructs are those where the indicators are effects of the underlying latent variable, while formative constructs are those where the indicators cause or form the latent variable. In our SEM model, socioeconomic status (SES) is conceptualized as a formative construct formed by various socioeconomic factors such as residency, house ownership, education, income, and other related variables. These formative indicators collectively define the SES construct, influencing oral health behaviors and outcomes. Structural equation modeling (SEM) was carried out using M-Plus V8.3 to test the hypothesized model based on a Petersen model (Fig. 1) [18]. While SES can be measured using objective indicators, this study conceptualizes SES as a latent variable to capture its multidimensionality, mitigate measurement error, and align with the theoretical framework of social determinants of health.

**Fig. 2:**
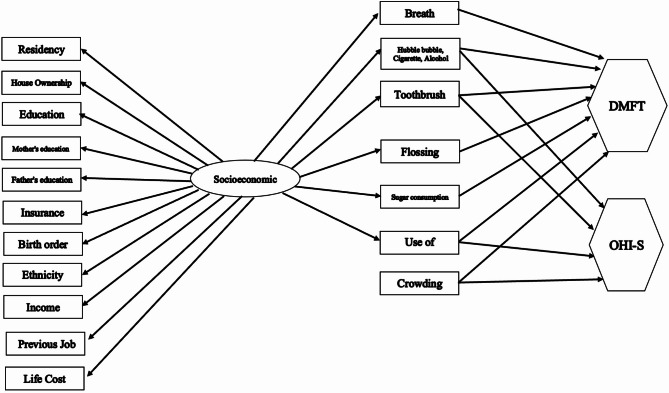
Structural Equation Modeling (SEM) shows the relationships between socioeconomic factors, oral health behaviors, and oral health outcomes. Socioeconomic status (SES) is modeled as a formative construct, indicated by multiple socioeconomic factors such as residency, house ownership, education, parental education, insurance, birth order, ethnicity, income, previous job, and cost of living. These indicators collectively form the SES latent variable, influencing oral health behaviors (e.g., breath, toothbrushing, flossing, sugar consumption) and oral health outcomes (DMFT and OHIS scores)

In SEM, observed variables are represented by rectangles, latent variables by ellipses, and outcomes by hexagons. Model fit was evaluated using the root mean squared error of approximation (RMSEA), Tucker-Lewis index (TLI), and comparative fit index (CFI), with acceptable limits defined as RMSEA < 0.1, CFI > 0.90, and TLI > 0.90 [19]. The initial model entered into the study is shown in Fig. 2.

Confirmatory factor analysis (CFA) was employed to assess the construct validity of socioeconomic status and environmental risk factors, which were treated as latent variables. Maximum likelihood estimation was used for the CFA. Convergent validity was assessed using standardized factor loadings, with loadings > 0.5 considered acceptable. Model fit indices were used to evaluate the model’s adequacy. Factors with standardized factor loadings below 0.5 were removed from the model as they did not demonstrate adequate convergent validity [19].

### Results

#### Demographic characteristics

The study sample consisted of 658 male soldiers, with a mean age of 21.2 ± 7.41 years. Most participants were single (94.5%) and resided in urban areas (68.2%). Regarding education, 14.3% had below diploma level, 46.8% had a diploma, and 38.9% had above diploma level. Detailed demographic information is presented in Table 2.

**Table 2:** Descriptive statistics of the study population (*n* = 658). frequency and percentage of background variables affecting oral Health among soldiers

Variable	Scale	Frequency	Percentage	Variable	Scale	Frequency	Percentage
Marital Status	Married	36	5.5%	Expenses status	Self-reported Financial Status	235	35.7%
	Single	622	94.5%		Family Financial Status	423	64.3%
Place of Residence	Urban	449	68.2%	Personal Income	No income	272	41.3%
	Rural	209	31.8%		Less than 1 million	276	41.9%
Education Level	Below Diploma	94	14.3%		More than 1 million	110	16.7%
	Diploma	308	46.8%	Family Income	Less than 1 million	118	17.9%
	Above Diploma	256	38.9%		Between 1 and 2 million	234	35.6%
Housing Status	Rented	118	17.9%		More than 2 million	242	36.6%
	Owned	540	82.1%	Dental Visit Frequency	No visits	251	38.1%
Father’s Education	Illiterate	137	20.8%		Emergency visits only	372	56.5%
					Regular checkups	35	5.3%
	Below Diploma	269	40.9%	Drug Use Habit	No Habit	473	71.9%
	Diploma	221	33.6%				
	Above Diploma	31	4.7%		Cigarettes or Qalyan	118	17.9%
Mother’s Education	Illiterate	176	26.7%		Alcohol	67	10.2%
	Below Diploma	286	43.5%	Brushing Habit	Irregular	263	40.0%
	Diploma	178	27.1%		At least once a day	395	60.0%
	Above Diploma	18	2.7%	Flossing Habit	No flossing	402	61.1%
Previous Insurance	None	156	23.7%		Irregular	216	32.8%
	Social Security	279	42.4%		At least once a day	40	6.1%
	Rural Insurance	96	14.6%	Sugary Food Consumption	Never	43	6.5%
	Other	127	19.3%		Once a day	255	38.8%
Previous Employment Status	Unemployed	270	41.0%		Twice a day	191	29.0%
	Employed	388	59.0%		More than twice a day	169	25.7%
Birth Order	Firstborn	212	32.2%	Importance of Oral Health	Very important	206	31.3%
	Secondborn	159	24.2%		Important	243	36.9%
	Thirdborn or higher	287	43.6%		Average	175	26.6%
Ethnicity	Persian	416	63.2%		Not important	34	5.2%
	Lur	107	16.3%	Gum Status	Healthy	152	23.1%
	Turk	38	5.8%		Inflamed	390	59.3%
	Kurd	7	1.1%		Receding	116	17.6%
	Arab	90	13.7%	Bleeding During Brushing	Yes	316	48.0%
Supplementary Insurance	Yes	17	2.6%		No	342	52.0%
	No	641	97.4%	Breathing Type	Mouth breathing	456	69.3%
Self-reported Oral Hygiene	Good	340	51.7%		Mouth and nose breathing	202	30.7%
	Average	271	41.2%	Crowding	Yes	443	67.3%
	Poor	47	7.1%		No	215	32.7%

#### Oral health status

The mean DMFT score was 3.57 ± 5.91, with the mean number of decayed, missing, and filled teeth being 2.39 ± 4.05, 0.75 ± 1.41, and 0.41 ± 1.10, respectively. The mean OHIS score was 0.56 ± 1.42, indicating a moderate level of oral hygiene among the participants.

#### Structural equation modeling

The structural equation model demonstrated a good fit, with a chi-square to degrees of freedom ratio of 31.2, RMSEA of 0.045, TLI of 0.892, and CFI of 0.915 (Table 3). The CFA results for socioeconomic status are shown in Table 4. Factors with loadings below 0.5 were removed from the model due to inadequate convergent validity. This resulted in excluding supplementary insurance, expenses incurred, and breathing patterns from the final model.

**Table 3:** Model fit indices were calculated using structural equation modeling

Fit index	Description	Reported value
CMIN	Chi-square	118.345
DF	Degrees of freedom	149
CMIN/DF	Chi-square/DF	31.2
TLI	Tucker-Lewis index	0.892
CFI	Comparative fit index	0.915
RMSEA	Root mean square error of approximation	0.045

**Table 4:** Estimated values, standard errors, and significance of factors on socioeconomic status in the modified model. confirmatory factor analysis was used to estimate factor loadings on socioeconomic status

Factor	Estimated value	Standard error	Significance
Living in the city	1.000	0.000	< 0.001
Homeownership	0.280	0.112	0.013
Individual’s education	0.477	0.108	< 0.001
Mother’s education	3.618	0.790	< 0.001
Father’s education	2.026	0.293	< 0.001
Previous insurance	0.271	0.089	< 0.001
Birth order	0.683	0.133	< 0.001
Ethnicity	0.432	0.103	< 0.001
Total	9.410	1.179	< 0.001

**Fig. 3:**
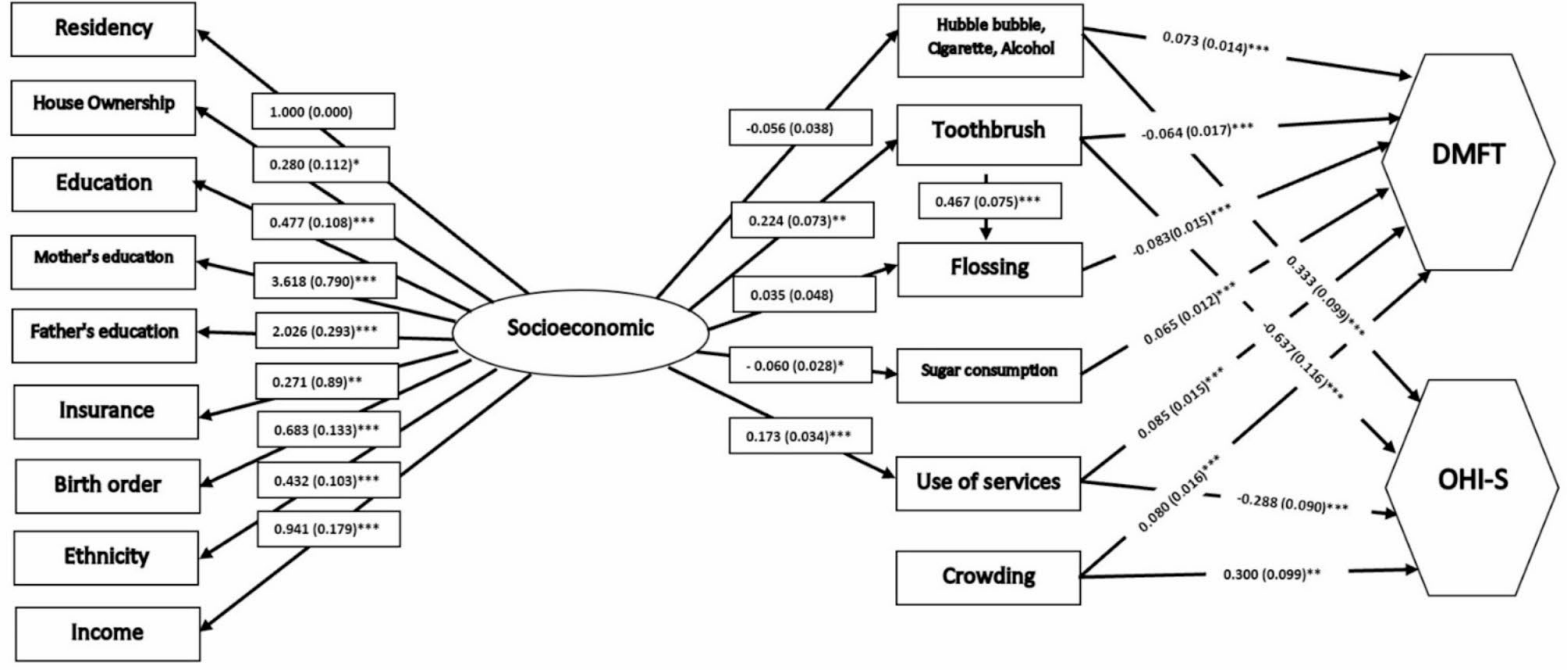
The final model for determinants of oral health, estimate (standard error). *p < 0.05; **p < 0.01; ***p < 0.001

The final model, depicting the relationships between socioeconomic status, oral health behaviors, and oral health outcomes, is shown in Fig. 3. Table 5 presents the standardized path coefficients (Estimated Values), standard errors, and significance levels for the relationships between the variables in the model.

**Table 5:** Standardized path coefficients (estimated values) and their associated standard errors and significance levels from the structural equation model

Factor	Estimated Value	Standard Error	Significance
Socioeconomic status of Drug use habit	-0.056	0.038	0.141
Socioeconomic status on brushing habit	0.224	0.073	0.002
Socioeconomic status on flossing habit	0.035	0.048	0.461
Socioeconomic status on sugary food consumption	-0.060	0.028	0.033
Socioeconomic status on dental visit frequency	0.173	0.034	< 0.001
Crowding on DMFT	0.080	0.016	< 0.001
Crowding on OHIS	0.300	0.099	0.002
Drug use habit on DMFT	0.073	0.014	< 0.001
Drug use habit on OHIS	0.333	0.099	0.001
Brushing habit on DMFT	-0.064	0.017	< 0.001
Brushing habit on OHIS	-0.637	0.116	< 0.001
Brushing habit on flossing habit	0.467	0.075	< 0.001
Flossing habit on DMFT	-0.083	0.015	< 0.001
Sugary food consumption on DMFT	0.065	0.012	< 0.001
Sugary food consumption on OHIS	0.012	0.082	0.886
Dental visit frequency on DMFT	-0.085	0.015	< 0.001
Dental visit frequency on OHIS	-0.288	0.090	< 0.001
Age on DMFT	0.114	0.025	< 0.001

As shown in Table 5, higher socioeconomic status was significantly associated with more frequent toothbrushing (Estimate = 0.224, *p* = 0.002), more frequent dental visits (Estimate = 0.173, *p* < 0.001), and lower consumption of sugary foods and drinks (Estimate = -0.060, *p* = 0.033).

Consistent with the SEM findings, lower toothbrushing frequency was significantly associated with higher DMFT scores (Estimate = -0.064, *p* < 0.001) and OHIS scores (Estimate = -0.637, *p* < 0.001). Soldiers who brushed their teeth at least once daily had significantly better oral health outcomes compared to those with irregular brushing habits. This demonstrates the importance of frequent toothbrushing in maintaining good oral hygiene and preventing dental caries.

Other notable findings include the significant association between daily toothbrushing and dental flossing (Estimate = 0.467, *p* < 0.001), suggesting that those who maintain positive oral hygiene behavior are more likely to engage in others. Additionally, regular dental flossing was independently associated with lower DMFT scores (Estimate = -0.083, *p* < 0.001), reinforcing the importance of interdental cleaning in preventing dental caries.

Similarly, more frequent dental visits were associated with lower DMFT scores (Estimate = -0.085, *p* < 0.001) and OHIS scores (Estimate = -0.288, *p* = 0.001) compared to those who only sought dental care for emergencies. Regular dental checkups provide opportunities for early detection and management of oral health issues, contributing to better long-term outcomes.

Interestingly, soldiers who reported not smoking, using qalyan, or consuming alcohol had lower DMFT scores (Estimate = 0.073, *p* < 0.001) and higher OHIS scores (Estimate = 0.333, *P* = 0.001) compared to those who engaged in these behaviors. This finding underscores the detrimental effects of drug use on oral health. Higher frequency of sugary food and drink consumption was significantly associated with higher DMFT scores (Estimate = 0.065, *p* < 0.001), indicating a detrimental effect of dietary sugar on oral health.

## Discussion

The present study used a structural equation modeling approach to investigate oral health status, caries experience, and associated factors among Iranian soldiers. The findings highlight the complex interplay between this population’s socioeconomic conditions, oral health behaviors, and oral health outcomes.

The mean OHIS score of 0.56 ± 1.42 found in this study indicates moderate oral hygiene among Iranian soldiers. Urban soldiers had lower OHIS scores compared to their rural counterparts, which may be attributed to disparities in oral health awareness and access to dental care. Only 17.3% of rural soldiers reported brushing their teeth at least once daily, compared to 42.7% of urban soldiers. These findings are consistent with previous studies that have reported significant differences in oral hygiene practices between urban and rural populations [20, 21].

Furthermore, soldiers who brushed at least once daily and had regular dental visits exhibited the lowest OHIS scores, emphasizing the importance of consistent oral hygiene practices and continuous dental care in maintaining good oral health. These results align with findings from studies conducted among military personnel in South Korea [22], Germany [23], and Malaysia [4], which highlighted the positive impact of frequent toothbrushing and regular dental attendance on oral hygiene status. However, the high proportion of soldiers who reported only seeking dental care for emergencies suggests that there are barriers to accessing preventive dental services. This finding is consistent with a study conducted among Thai military hospital personnel, which reported a high prevalence of dental diseases, with over 98% requiring dental treatment and only 1.2% not needing dental care [24]. Addressing these barriers and promoting regular dental checkups should be a priority for military health authorities.

The mean DMFT score of 3.57 ± 5.91 found in this study is lower than those reported in previous studies among military personnel in Iran [7], Malaysia [25], Jordan [26], and Croatia [5]. However, it is higher than the global average DMFT for adults aged 35–44 years, which is 2.11 [3]. This difference can be attributed to the younger age of the study population in the present study, as increasing age is strongly correlated with a higher prevalence and incidence of dental caries [27–30]. This difference from the global average may be attributed to the unique challenges soldiers face, such as demanding physical and psychological training, inconsistent hygiene practices, and limited access to dental care [5].

The structural equation model employed in this study assessed the direct and indirect factors influencing oral health status. Socioeconomic conditions indirectly influenced DMFT and oral hygiene through mediating factors such as drug use, oral hygiene practices, dietary sugar consumption, and dental visits. The formative nature of SES means that it is constructed from multiple observed variables (residency, house ownership, education, etc.), which collectively define the SES of an individual. These SES indicators impact oral health behaviors and outcomes, illustrating the complex interplay between various determinants of oral health. These findings are consistent with previous studies showing a link between health behaviors, including oral health practices, and socioeconomic factors [10, 31–33].

Higher education levels and better parental education, as indicators of socioeconomic status, were associated with increased awareness of the harmful effects of sugary foods and drinks, more frequent dental visits, and better individual oral hygiene practices, which have been linked to better oral health outcomes in previous studies [18, 34, 35]. Our findings regarding the association between lower education levels and poorer oral health align with observations by Senna et al. Their study on Italian conscripts and military academy students also found a higher prevalence of caries among those with lower education levels, highlighting the consistent impact of education on oral health across different military populations [36].

Smoking, alcohol consumption, and qalyan use were significantly associated with higher DMFT and OHIS scores in this study. As young adults separated from their families and facing various health challenges, soldiers are particularly vulnerable to drug use and may neglect their health, including oral hygiene. These findings are consistent with previous studies that have reported the detrimental effects of smoking and other drug use on oral health among military personnel [7, 8, 37]. Implementing comprehensive health promotion programs that address these risk factors and provide cessation support should be integral to military health initiatives.

The strong association between sugary food consumption and oral health found in this study aligns with the well-established link between dietary sugar intake and dental caries [38, 39]. More than half of the soldiers in this study consumed sugary foods more than once daily, emphasizing the need for targeted interventions to promote healthier dietary habits in this population.

In recent decades, Iran has implemented several public health programs to improve oral health. For instance, a comprehensive study by Pakshir (2004) highlighted the success of the national oral health promotion program in schools, which led to a significant reduction in DMFT scores among 12-year-olds, from 4 in 1988 to 1.5 in 2004 [40]. The 2015 Oral Healthcare Reform by the Ministry of Health and Medical Education also promoted oral health in children under age 14 at the national level. This reform aimed to enhance the availability and quality of oral health services, emphasizing preventive care and early intervention [41]. Additionally, Iran has integrated oral health care with primary health care as a leading strategy for accessing oral health care services for children and adolescents, including fluoride therapy, dentist visits, and caries prevention. However, the implementation of oral health policies has encountered several challenges. Expanding national coverage of oral health services, integrating these services into preventive care, and focusing on the private sector are likely the most critical strategies for improving oral health outcomes in Iran [41, 42]. These programs have not yet significantly addressed the issue of poor oral health among soldiers. The lack of adequate insurance coverage, limitations of public dental care systems, disparities in access to dental care, and high costs of dental services for the majority of the population are major contributing factors, along with cultural factors [9, 43].

The strengths of this study include its comprehensive examination of factors influencing oral health, including socioeconomic factors, using standardized indices such as DMFT and OHIS within a conceptual model. Structural equation modeling allowed for the simultaneous analysis of multiple variables and their interrelationships, providing a more holistic understanding of the factors contributing to oral health status in Iranian soldiers. However, the study also has some limitations. The study’s cross-sectional design precludes the establishment of causal relationships between the studied variables. Furthermore, our study’s cross-sectional design limits our ability to assess the influence of time spent in military service on oral health. The self-reported nature of some data, such as oral hygiene practices and dietary habits, may be subject to recall and social desirability bias.

This study focuses on male soldiers because military service is not for women in Iran. Additionally, studying a homogeneous gender group allowed us to control for potential gender-related confounding factors in oral health status and behaviors. Future studies should explore oral health determinants among women in the military as their roles and experiences may differ. Moreover, while our study acknowledges the unique stressors and lifestyle factors inherent to military service, such as demanding training schedules, potential dietary imbalances, and psychological stress, we did not directly investigate these aspects and their specific impact on oral health outcomes within our sample. Future studies should explore these factors in more detail to provide a more comprehensive understanding of oral health determinants in this population. Also, future research should focus on longitudinal studies to better understand the causal pathways between socioeconomic, behavioral, and environmental factors and oral health outcomes in military populations over time. Qualitative studies exploring the barriers and facilitators to accessing dental care services and adopting positive oral health behaviors among soldiers could provide valuable insights for developing targeted interventions.

## Conclusion

This study provides a snapshot of the oral health status and caries experience among Iranian soldiers, highlighting the significant impact of socioeconomic factors such as drug use, oral hygiene practices, dietary sugar consumption, dental visits, and access to dental care. Our findings underscore the importance of a multifaceted approach to improving oral health outcomes in this population. Specifically, targeted interventions should promote regular dental checkups, improve oral hygiene practices through educational initiatives, and address the socioeconomic barriers that hinder access to preventive dental care. Additionally, addressing the high prevalence of drug use within this population and its detrimental effects on oral health should be prioritized. By implementing comprehensive oral health promotion programs that consider the unique challenges faced by soldiers, military health authorities can significantly improve the oral health and overall well-being of this important demographic.

## Electronic supplementary material

Below is the link to the electronic supplementary material.

Supplementary Material 1

## Data Availability

The datasets used and analyzed during the current study are available from the corresponding author on reasonable request.

## Abbreviations

DMFT: Decayed, Missing, and Filled Teeth
OHIS: Simplified Oral Hygiene Index
SEM: Structural Equation Modeling
SES: Socioeconomic Status
WHO: World Health Organization
CFA: Confirmatory Factor Analysis
RMSEA: Root Mean Square Error of Approximation
TLI: Tucker-Lewis Index
CFI: Comparative Fit Index
