# Supplementary material for: Determinants of oral health among Iranian soldiers: a structural equation modeling study
Source: BMC Oral Health. 2024 Oct 25;24:1288. doi: 10.1186/s12903-024-05052-5 (PMC11515288; doi:10.1186/s12903-024-05052-5)
Supplement: Supplementary file 1 — Supplementary Material 1 [file 12903_2024_5052_MOESM1_ESM.pdf]

Oral hygiene instrument (English version) **Code:**

Period of service:
Month: ….. Day: …….

The place of service:
……………………………………

Place of Residence:
City............................ Village……….....................

Education:
Individual's: 1. Below Diploma □ 2. Diploma □ 3. Above Diploma □
Mother's: 1. Below Diploma □ 2. Diploma □ 3. Above Diploma □
Father's: 1. Below Diploma □ 2. Diploma □ 3. Above Diploma □

Previous employment status:
1. Unemployed □
2. Employed □

Age:

Previous Insurance:
1. None □ 2. Social Security □ 3. Rural Insurance □ 4. Other ………
Supplementary Insurance: 1. Yes □ 2. No □

Housing Status:
Owner □ Rental □

Ethnicity: Persian□ Lur□ Turk□ Kurd□ Arab□

Birth Order: 1. Firstborn □ 2. Secondborn □ 3. Thirdborn or higher □

Marital status:
1. Single □ 2. Married □

Who is the source of your payment? 1. Yourself □ 2. Family □

Monthly personal income (Tomans): 1. No income □ 2. Less than 1 million □ 3. More than 1 million □
Monthly the family income (Tomans): 1. Less than 1 million □ 2. Between 1 and 2 million □ 3. More than 2 million □

Dental Visit Frequency: 1. No visit □ 2. Emergency visits only □ 3. Regular checkups □

Medical History and Systemic Disease:
1. Gastrointestinal disease □ 2. Respiratory disease □ 3. Blood diseases □ 4. Allergy □
5. Diabetes □ 6. Cardiovascular disease □ 7. Paralysis or disability □ 8. Epilepsy □
9. Mental illness □ 10. Kidney disease □ 11. Nervous disease □ 12. Others: …………
The Name of Medicine used: ………………………………………………………………………………………………..

Oral hygiene habits:
Brushing Habit: 1. Irregular □ 2. At least once a day □
Flossing Habit: 1. No flossing □ 2. Irregular □ 3. At least once a day

Drug Use habits:
1. No Habit □ 2. Cigarettes & Qalyan □
3. Alcohol □

In your opinion, how is your oral health? 1. Good □ 2. Average □ 3. Poor □

The following parts should be completed by the dentist

Frequency of consumption of sugary substances per day: 1. Never □ 2. Once □ 3. Twice □ 4. More than twice a day □

How much do you care about your oral health problems? 1. Very important□ 2. Important□ 3. Average□ 4. Not important□

Breathing Type: 1. Mouth breathing □ 2. Mouth and nose breathing □

Dental crowding: 1. Yes □ 2. No □

The condition of the patient's teeth (DMFT): 1. Number of decayed teeth: .... 2. Number of filled teeth: ....
3. The number of missing teeth: ....

Gum status: 1. Healthy □ 2. Inflamed □ 3. Receding □

Bleeding during brushing: 1. Yes □ 2. No □

Oral health index (OHIS):

Debris Index (DI)

	Right molar		Front area		Left molar		Total	
	Buccal	Lingual	Labial R	Labial L	Buccal	Lingual	Buccal	Lingual
Max								
Man								

Calculus Index (CI)

	Right molar		front area		Left molar		Total	
	Buccal	Lingual	Labial R	Labial L	Buccal	Lingual	Buccal	Lingual
Max								
Man								

* This form is designed to check oral health and related factors in soldiers. Its information is completely confidential and participation is optional.
